# Model-based image analysis of a tethered Brownian fibre for shear stress sensing

**DOI:** 10.1098/rsif.2017.0564

**Published:** 2017-12-06

**Authors:** M. T. Gallagher, C. V. Neal, K. P. Arkill, D. J. Smith

**Affiliations:** 1 School of Mathematics, University of Birmingham, Birmingham B15 2TT, UK; 2 Institute for Metabolism and Systems Research, University of Birmingham, Birmingham B15 2TT, UK; 3 Centre for Human Reproductive Science, Birmingham Women's and Children's NHS Foundation Trust, Birmingham B15 2TG, UK; 4 School of Medicine, University of Nottingham, Nottingham NG7 2UH, UK; 5 Biofisika Institute (CSIC UPV/EHU), University of the Basque Country, 48080 Bilbao, Spain; 6 Research Centre for Experimental Marine Biology and Biotechnology, University of the Basque Country, 48080 Bilbao, Spain

**Keywords:** image analysis, wall shear stress, Brownian dynamics, regularized stokeslets, mathematical modelling, viscous fluid dynamics

## Abstract

The measurement of fluid dynamic shear stress acting on a biologically relevant surface is a challenging problem, particularly in the complex environment of, for example, the vasculature. While an experimental method for the direct detection of wall shear stress via the imaging of a synthetic biology nanorod has recently been developed, the data interpretation so far has been limited to phenomenological random walk modelling, small-angle approximation, and image analysis techniques which do not take into account the production of an image from a three-dimensional subject. In this report, we develop a mathematical and statistical framework to estimate shear stress from rapid imaging sequences based firstly on stochastic modelling of the dynamics of a tethered Brownian fibre in shear flow, and secondly on a novel model-based image analysis, which reconstructs fibre positions by solving the inverse problem of image formation. This framework is tested on experimental data, providing the first mechanistically rational analysis of the novel assay. What follows further develops the established theory for an untethered particle in a semi-dilute suspension, which is of relevance to, for example, the study of Brownian nanowires without flow, and presents new ideas in the field of multi-disciplinary image analysis.

## 1. Introduction

The force per unit area exerted on a surface by a moving fluid, otherwise known as wall shear stress (WSS), plays an important role in many physical and biological systems, for example the function and structure of endothelial cells [1,2] and the design of microfluidic systems [3,4]. While there exist several ways of measuring WSS directly [5–7], these methods are not suitable for measuring WSS in, for example, the vasculature, as they either require insertion of deformable micropillars (approx. 100 μm tall) or neglect to take into account biologically relevant aspects of the flow, for example the pulsatile nature of the flow in the vasculature which also contains fluid particulates and has complex geometries. There are also other biological factors limiting such flow methods; the viscosity of many fluids of interest is often not known, and can change with time, introducing additional error into calculations. We also know that cell surface macromolecules (for example the glycocalyx) can extend a distance > 0.5 μm into the fluid, meaning that surface effects become important and difficult to calculate. The current method for measuring WSS in the vasculature relies on measurement of the velocity gradient on the wall through bulk flow techniques such as micro-particle image velocimetry (μPIV) [8–10]. However, due to the size of the particles needed to measure flow through blood vessels, Brownian effects become important which can introduce error in the measurement of velocities and uncertainty in the location of the particles. In this research, we turn the Brownian motion of particles to our advantage; instead of needing to correct for such effects, the Brownian motion of a tethered rod is the measurement mechanism which underpins this work.

To measure shear stress in the vasculature at the same place that an endothelial cell can detect requires a sensor that can respond to shear stress in the same location. We continue the development of a sensor that can detect shear stress in microvessels as close as a few hundred nanometres from the cell membrane in real time in live animals. A biological microrod approximately 1 μm in length, based on M13 bacteriophage (hereafter referred to as M13), has recently been demonstrated to act as such a surface shear stress sensor [11] through flow-induced changes to its tethered Brownian motion. The M13 is 7 nm wide and ≈ 900 nm long, with a persistence length of 1265.7 ± 220.4 nm [12]. It forms a semi-rigid ‘nanorod’ which can be genetically engineered, or chemically modified to bind to fluorescent moieties or antibodies. These monodisperse nano-particles have been used to produce several nanoscale devices including nanowires [13,14] and scaffolds for polymerase chain reaction [15]. Other methods using orientations of freely suspended nanorods have been employed by Kim *et al.* [16], where the real-time measurement of the collective orientation of nanorods has been used to measure local shear rate in microfluidic systems. The collective orientation of suspensions of nanorods has also been used to detect pathogenic bacteria through the shear alignment of virus particles and linear dichroism by Pacheco-Gómez *et al.* [17]. These characteristics have been used to generate an M13 construct that includes a collagen antibody covalently attached to one end, and decorated with more than 500 fluorophores along its length. This construct allows the M13 to bind at one end to a collagen-coated slide, and be imaged using epi-fluorescent microscopy. It is this construct that we will focus on in this report.

The framework for the modelling and measurement of WSS constructed in this report consists of two key steps: modelling the dynamics of a tethered Brownian fibre, and the extraction of experimental data through the use of *model-based image analysis*. The modular nature of this framework will mean that it can be easily extended to investigate related problems in both microscale biology and areas where reliable, rational analysis of experimental image data is desired. In what follows, we simplify the problem by approximating the M13 to be a stiff thin rod. This is a rational first approximation as the M13 in the associated experiments of Lobo *et al.* [11] has lengths below their associated persistence length, as experimentally measured by Khalil *et al.* [12].

Under no flow, the attached M13 oscillates randomly due to Brownian motion. As a flow is applied, however, the M13 movement is biased towards the direction of flow. This biasing behaviour is characterized through calculation of the Péclet number, the ratio between Brownian and advective effects due to shear. Brownian rotation is inversely proportional to viscosity, and advective rotation is proportional to shear rate. Therefore, the Péclet number is proportional to the product of the shear rate and viscosity, i.e. the shear stress. Knowledge of the Péclet number, combined with temperature and phage geometry, thereby yields the shear stress (knowledge of the viscosity also then yields the shear rate, and vice versa). Data interpretation has so far been limited to phenomenological random walk modelling, and small-angle approximation to the resulting partial differential equations; however, to apply the M13 quantitatively and to assess effects such as surface topography and variations in fibre length, it is valuable to model the underlying fluid dynamics of the tethered rod. We develop a mathematical framework for the rotational Brownian dynamics of a tethered M13, using rational mechanistic modelling to gain deep understanding about the behaviour of the M13 and its relationship to WSS. What follows is relevant to the established theory for an untethered particle in a semi-dilute suspension [16,18], and also to, for example, the recent study of Brownian nanowires without flow by Ota *et al.* [19].

Owing to the width of the M13 (7 nm) being much smaller than the wavelength of light used to excite the attached fluorophores (561 nm) the produced image is heavily diffracted and as such it requires work to calculate the exact location of the M13. Traditionally, deconvolution algorithms would be applied to such an image, either with *a priori* knowledge of how the light has been diffracted or without (blind deconvolution); several such schemes are available as packages in both ImageJ [20] and Matlab [21] as well as others. Current methods to do this often involve the use of ‘black box’ processing algorithms. While these tools can be useful, and often provide good information, a lack of transparency can hinder interpretation, particularly in a context where statistical properties of the error are crucial, and as such can never give complete confidence in the results. Even when the details of such algorithms are known, they often rely on changing the image without any knowledge of what the image contains or how it was formed. To combat this, we develop here the concept of *model-based image analysis*. Using knowledge of the physics of image formation, including understanding of how optical effects such as diffraction of light occur, we construct a mathematical framework for the inverse problem of image formation: how, given an experimental image, we can calculate what originally formed the image by undoing the image formation process. Besides providing a rational framework for analysing images, model-based image analysis produces consistent results and can be applied to any experimental set-up where the knowledge of the image formation is sufficiently well understood.

In this report, we combine work from the areas of synthetic biology and mathematical modelling, together with fluid dynamics and the concept of model-based image analysis to create a framework for the measurement of WSS in biological systems. In the first part of this work, we present the dynamics of a tethered Brownian fibre, and relate the angle distribution of the M13 in flow to the Péclet number, the ratio between Brownian and convective effects in the flow. We continue by introducing the concept of model-based image analysis and the inverse problem of image formation, and include algorithms for the automated processing of the experimental image data. The automated nature of the image processing, and its high throughput of data, enables the accuracy of the methods to be analysed through large-scale simulations of data. Finally, we combine all these ideas to calculate the WSS for the flow. The principle will then be demonstrated on the experimental data of Lobo *et al.* [11], providing the first mechanistically rational analysis of this novel assay.

## 2. Dynamics of a tethered Brownian fibre

**Figure 1.**
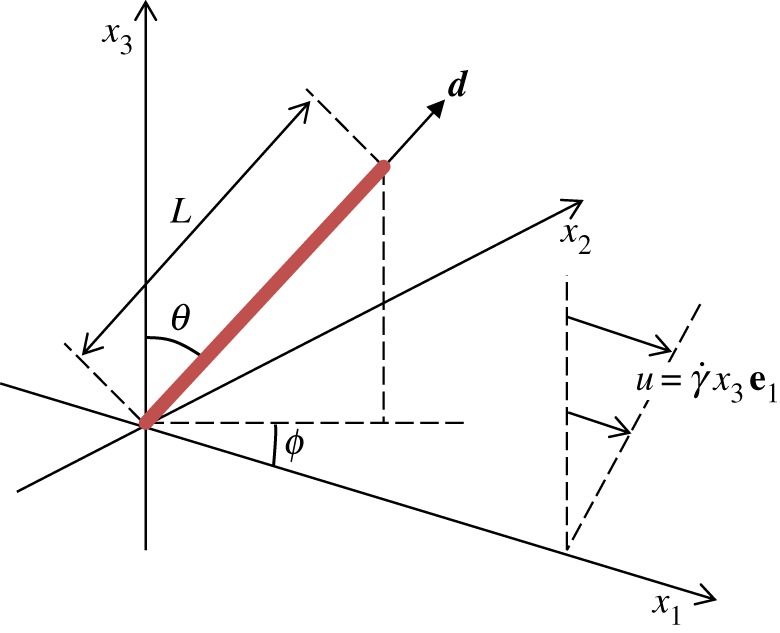
Definition sketch showing the location of the M13 (red), along with the direction of the applied shear flow. Here the M13 is tethered at (0, 0, 0) to the solid (*x*_1_, *x*_2_)-plane, with direction vector ***d***.

We model the rotational Brownian dynamics of a rigid axisymmetric fibre of length *L* projecting into the half-space *x*_3_ > 0, attached at (0, 0, 0) to the solid plane boundary *x*_3_ = 0 under homogeneous unidirectional shear flow 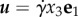. A definition sketch is included in figure 1. This choice of flow and geometry will provide a strong basis upon which these methods can be extended to reflect other interesting biological problems. Working in spherical polar coordinates (*r*, *θ*, *ϕ*) and following Kim & Karrila [22], we define ***d***(*θ*, *ϕ*) to be the direction vector of the M13, with the triple 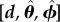 being the basis vectors. We denote by **∇**_***d***_ and **∇**_***d***_ · the angular parts of the spherical polar gradient and divergence operators,2.1
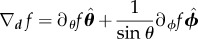
and2.2
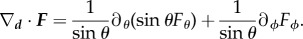
The problem will be to determine the steady state of the probability density function *ψ*(*θ*, *ϕ*, *t*) for the fibre orientation, on the unit hemispherical domain 0° ≤*θ* ≤90° and 0° ≤*ϕ* < 360°.^1^ The probability density will satisfy the normalization condition2.3
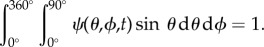
Note the change relative to [18,22] in the absence of the 4*π* factor in equation (2.3), so that the unscaled *ψ* is a probability density function (the factor of 4*π* is less appropriate when working on a hemispherical domain). Two-dimensional imaging will directly yield a projection onto the (*x*_1_, *x*_2_)-plane, so we will observe samples from the marginal density function,2.4
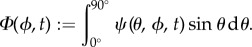

The flux vector of *ψ* in (*θ*, *ϕ*) space is given by 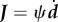, where 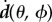 is the rate of change of ***d*** due to the combination of hydrodynamic and Brownian rotations. After some work, we obtain the advection–diffusion equation2.5
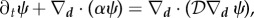
where 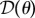 is the rotational diffusion matrix and ***α***(*θ*, *ϕ*), the rate of change of ***d*** under rigid body rotation, is the rotational advection vector. Details of the derivation of (2.5) are given in appendix A. Introducing dimensionless variables *t*′, 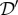, ***α***′, we have2.6
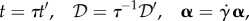
with characteristic time scale *τ* =*μL*^3^/*kT*, where *k* is the Boltzmann constant and *T* is the absolute temperature. The dimensionless advection–diffusion equation is then2.7
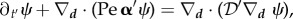
where the rotational Péclet number 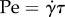. It is important to note the inclusion of the shear rate 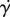 in this definition of the rotational Péclet number; in what follows, we will use the measurement of Pe as a proxy for measurement of shear. In the current work, we make the assumption that *ψ* is independent of time for a given flow (for a fixed Péclet number), which gives the steady-state dimensionless advection–diffusion equation2.8
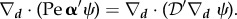
The coefficients 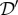 and ***α***′ will be calculated by solving the dimensionless rotational resistance and mobility Stokes flow problems, respectively, after which the probability density function *ψ* can be calculated by solving (2.8) subject to the normalization condition (2.3). We solve (2.8) directly using a centred finite difference scheme in Matlab [21]. The full expression for (2.8) is given in appendix B.

### 2.1. Solution of the rotational resistance and mobility Stokes flow problems

There exist several approaches to solving the resistance and mobility Stokes flow problems, including finite element, boundary integral and regularized stokeslet methods, in addition to approximations based on slender body theory. In this paper, we apply a novel variation on the method of regularized stokeslets, namely the nearest-neighbour discretization of Smith [23]. This method retains the ‘meshlessness’ of the original formulation, with the added benefit of having a major reduction in computational cost.

The small Reynolds number associated with microscale flow justifies the use of the (dimensionless) Stokes flow equations,2.9
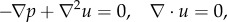
where *p* is the pressure and ***u*** is the velocity. The relevant boundary conditions are no-slip/no-penetration on the plane ***u***(*x*_1_, *x*_2_, 0, *t*) = 0, no-slip/no-penetration on the rigid body 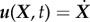, and convergence to a prescribed steady far-field flow 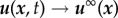 as 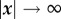.

A solution to equation (2.9) with the given boundary conditions may be expressed as a regularized stokeslet boundary integral,2.10
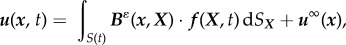
where *S*(*t*) denotes the body surface, *f*_*k*_ the hydrodynamic force per unit area exerted by the body on the fluid and *B*^*ɛ*^_*jk*_ the regularized ‘blakelet’ found by Ainley *et al.* [24],2.11
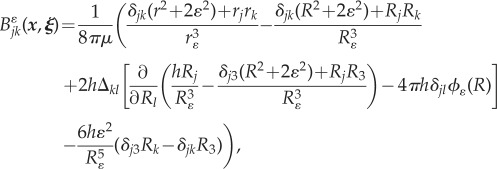
where *ɛ* is a small regularization parameter, taken to be 1% of the M13 length. Imposing the boundary conditions on the surface of the body, along with rigid body rotations about the origin, we have the first kind Fredholm integral equation for the unknown force density ***f***(***X***, *t*) at each instant *t*, namely2.12
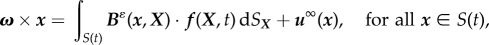
with *ω* being the rotational velocity of the M13, and ***B***^*ɛ*^(***x***, ***X***) being a kernel which is large but finite when ***x*** = ***X***. In the inertialess regime, the system is closed by specifying the torque on the body due to hydrodynamic stress,2.13
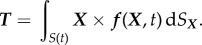


The *mobility problem* for this set-up then corresponds to the system of equations (2.12) and (2.13) with ***T*** and ***u***^∞^ prescribed and ***ω*** unknown. The *resistance problem* corresponds to the same system with ***ω*** and ***u***^∞^ prescribed and ***T*** unknown.

The dimensionless rotational advection vector ***α***′ is then given by solving the mobility problem for 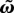, prescribing ***T*** = 0 (corresponding to zero applied torque) and ***u***^∞^ = *x*_3_**e**_1_ (corresponding to unit shear flow). Then we have that 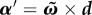. Recall that ***α***′ = ***α***′(*θ*, *ϕ*); therefore, it is necessary to find an approximate solution over the domain (*θ*, *ϕ*) ∈ [0°, 90°] × [0°, 360°).

The dimensionless diffusion coefficient 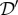 is given by solving the resistance problems for ***T***_*θ*_′ and ***T***_*ϕ*_′ , prescribing, respectively, ***ω*** = ***e***_*θ*_ and ***ω*** = ***e***_*ϕ*_ (corresponding to the two rotational modes), along with zero incident flow ***u***^∞^ = 0. Once these torques are found, the dimensionless resistance matrix in (*θ*, *ϕ*) coordinates can be assembled as 
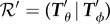
 the dimensionless diffusion coefficient is then 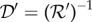. Recall that 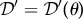; an approximate solution must, therefore, be found for all *θ* ∈ [0°, 90°], where, without loss of generality, we can set *ϕ* = 0°.

### 2.2. Numerical results

**Figure 2.**
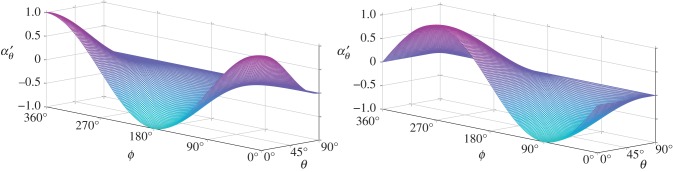
Components of the dimensionless rotational advection vector ***α***′ plotted for 0°≤ *ϕ* < 360° and 0°≤ *θ* ≤ 90°.

**Figure 3.**
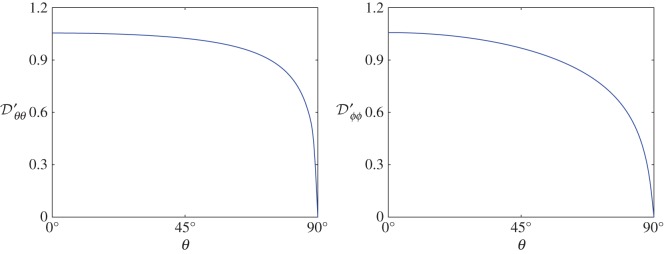
Non-zero components of the dimensionless diffusion matrix 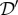 plotted against 0°≤ *θ* ≤ 90°.

The dimensionless rotational advection vector ***α***′ is solved over a grid with 0° ≤*θ* ≤90° and 0° ≤*ϕ* ≤360°, and is then interpolated using a cubic spline with periodic end conditions at the *ϕ* limits. The resulting components *α*_*θ*_, and *α*_*ϕ*_ are shown in figure 2. Similarly, the dimensionless rotational diffusion matrix 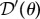, solved over 0° ≤*θ* ≤90°, is again interpolated using a cublic spline, and is shown in figure 3. In solving for 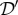 numerically, we have introduced a small regularization, at *θ* = 90°, through enforcing 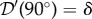 (in our calculations, we use *δ* = 0.01). This ensures that the solutions for 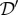 remain regular as 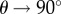. Finally, the advection–diffusion equation (2.8) is solved for 1 ≤ Pe ≤ 200. Here, the bounds on Pe have been chosen to include the experimentally relevant range for this project, but could be changed depending on the problem at hand.

**Figure 4.**
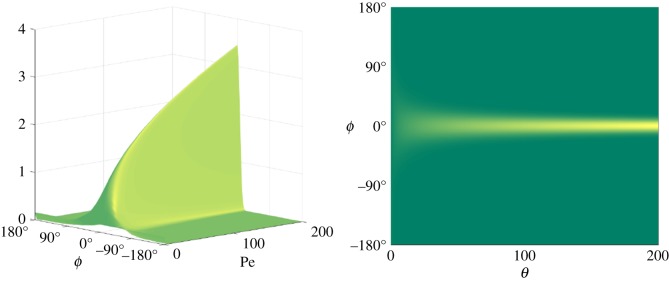
Marginal probability density function *Φ*, the steady solution to the advection–diffusion equation (2.4), plotted with −90°≤ *ϕ* ≤ 90° and 1 ≤ Pe ≤ 200.

The marginal probability density function *Φ* (2.4) is obtained by integrating *ψ* over 0°≤ *θ* ≤ 90°; the result is shown in figure 4. As expected, we see that the larger the Péclet number, the more likely the M13 is to be aligned in the direction of the flow. Also as expected, when 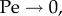 we see the biasing effect decreases rapidly with the M13 approaching a uniform distribution. This behaviour is consistent with the physical interpretation of the Péclet number, with the case Pe = 0 describing purely Brownian dynamics, with large Péclet numbers corresponding to shear dominated flows. Having calculated *Φ* for a range of Pe, we should now be able to estimate Pe for a given set of angles *ϕ*. The methods by which we do this will be discussed in §3.2.

In order to measure the Péclet number in a biological system given the theory presented above, we require methods for the extraction of orientation data from experimental images. To this end, we now turn our attention to developing the concepts of model-based image analysis.

## 3. Detection of a tethered Brownian fibre

Having established a mathematical model for the dynamics of a tethered Brownian fibre, we now turn our attention to the application of the model to the experimental data of Lobo *et al.* [11], with a view to calculating the Péclet number for an applied shear flow. The experimental procedure for obtaining images of the tethered M13 is contained within [11] and as such is not repeated here, except for noting that the experimental set-up was that of a fluorescently labelled M13 tethered to a collagen-coated slide which was then imaged with a 1.4NA oil objective with a spinning disc confocal microscope (Ultraview; PerkinElmer). In what follows, we attack the problem through novel mathematical model-based image analysis methods which, along with the theory presented in §2, will provide a more rigorous and extensible basis for future work.

### 3.1. The inverse problem of image formation

We model the experimental M13 as a rigid, inextensible, axisymmetric rod of length *L* projecting into the half-space *x*_3_ ≥ 0. The M13 is tethered at the point (*x*_0_, *y*_0_, *z*_0_) of the Cartesian coordinate system (*x*_1_, *x*_2_, *x*_3_) to the solid plane boundary *x*_3_ = 0, and is subjected to homogeneous unidirectional shear flow 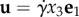. The position of the M13 (*x*_1_, *x*_2_, *x*_3_) = (*x*, *y*, *z*) is then given, in spherical polar coordinates, as3.1
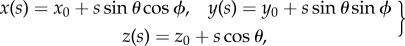
for given azimuthal and polar angles 0° ≤*θ* ≤ 90° and 0° ≤*ϕ* < 360°, with 0 ≤*s* ≤ *L* being the arclength along the M13. See figure 1 for a sketch of the set-up noting that, in what follows, we now model the M13 as being tethered to some, as yet, unknown point (*x*_0_, *y*_0_, *z*_0_).

Following Zhang *et al.* [25], we model the optical diffraction of a light source located at the point (*X*_0_, *Y*_0_, *Z*_0_), diffusing over the focal plane (*X*, *Y*, *Z*), by a Gaussian point spread function (PSF), namely3.2
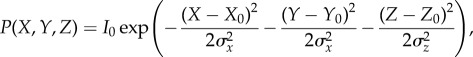
where *I*_0_, *σ*_*x*_ and *σ*_*z*_ are parameters relating to the experimental set-up. Note that we have assumed that the optical diffraction will be equal in both the **e**_1_ and **e**_2_ directions when imaged from above, resulting in a circular PSF for a given focal plane *z* = *z*_0_. The resulting image, *I*, given by convolution of the PSF (3.2) with the M13 location (3.1), in the focal plane (*x*_1_, *x*_2_, 0), is then3.3
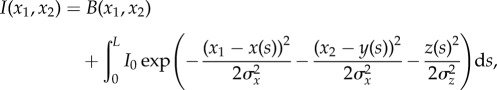
where *B* is some background image intensity, which may be constant or may vary with pixel location.

Given a set of experimental images, and a model for the forward problem of image formation (3.3), it remains to solve the inverse problem of image formation: estimation of the position of the M13 given an experimental image. In order to ensure a good fit between the experimental and simulated images, we choose the intensity parameter *I*_0_ to be3.4
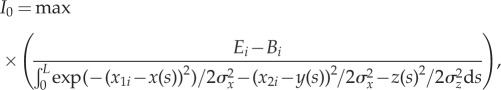
over all *i* pixels in the image. We define the M13 location to be the set of spatial parameters (*x*_0_, *y*_0_, *ϕ*, *θ*), and optical parameters (*I*_0_, *σ*_*x*_, *σ*_*z*_ and B) which minimize the sum-squared error between the experimental and simulated images, namely3.5
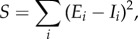
where *E*_*i*_ and *I*_*i*_ are the *i*th pixels in the experimental and simulated images, respectively. Note that the intensity of the *i*th pixel in the image *I*_*i*_, with index *i* ≥ 1, should not be confused with the PSF intensity *I*_0_. The minimization is performed globally using the multi-level coordinate search algorithm, routine *e05jb*, from the NAG Toolbox for Matlab [26], with a set of bounds on each of the parameters. Owing to the complexity of the problem, and the lack of detailed information regarding the optical parameter *σ*_*z*_, in what follows we model each image as though it contains an M13 of variable projected length *L*, inclined at an angle *θ* = 90° to the vertical. Here, we constrain the M13 parameters through requiring (*x*_0_, *y*_0_) to lie within the image, 0 < *L* < 2 μm and −90° ≤*ϕ* ≤90°. We then require that the optical parameters have the following constraints: 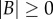 and 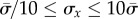, where 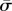 is given by following Zhang *et al.* [25].

### 3.2. Fitting procedure

In refining our fitting algorithms we found that a small amount of preprocessing of the experimental images led to a significant increase in the accuracy of the fits. The preprocessing step involves applying a 5 × 5-pixel median filter [27] to the experimental image, followed by subtracting the median image intensity from all pixels in the image, and finally setting the values of all pixels with negative intensity to zero. The effect of this preprocessing step is analysed in §4. We then perform a multi-stage fit in order to find the M13 and optical parameters which can best replicate the given experimental image as follows.

— We first fit the spatial parameters for initial optical parameters *σ*_*x*_ and *B*. Owing to the preprocessing of the experimental images, we choose *B* = 0. The PSF spread *σ*_*x*_ is approximated by following Zhang *et al.* [25] for the experimental set-up.
— Having calculated a first guess for the spatial parameters, the value for *σ*_*x*_ is then fitted, keeping all other parameters fixed. While, theoretically, the value of *σ*_*x*_ should be constant for all images from a given experiment, due to the preprocessing step, we allow some variation in *σ*_*x*_ to take place.
— The spatial parameters are now refitted using the updated value for *σ*_*x*_.
— We then fit the image background *B*, while allowing a small change in *σ*_*x*_ if necessary.
— Finally, the new values of *σ*_*x*_ and *B* are used to fit the spatial parameters (*x*_0_, *y*_0_, *L*, *ϕ*).

Once the M13 and optical parameters have been obtained for all the experimental images, we can use the theory discussed in §2 to estimate the Péclet number for a particular flow. Using the marginal probability density function for the flow *Φ* (shown in figure 4), we can integrate to find the related cumulative density function (CDF) *F*_1_, which can then be compared with the sample CDF *F*_2_ through calculation of the Kolmogorov–Smirnov statistic *D* [28],3.6
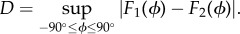
The Péclet number 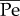 that minimizes *D* is then chosen as the fit. This optimization procedure is again done with the multi-level coordinate search algorithm (*e*05*jb*) from the NAG Toolbox for Matlab [26]. The accuracy of the fitting procedures is now investigated.

## 4. Accuracy of fluid dynamics modelling with model-based image analysis

In order for this model-based image analysis framework to be useful, it must be able to accurately fit the location of a series of M13, and the Péclet number corresponding to the flow over such M13. We investigate the accuracy of the fit by dividing the problem into two areas where error can be introduced, namely the image processing stage, and the calculation of the Péclet number from a sample of orientation data. For each of these steps, we will generate 180 sample images for a spread of Péclet numbers 1 ≤Pe ≤200, which is comparable to both the number of images and the flow rates of the associated experiments.

### 4.1. Step 1: error associated with image processing

In investigating the error associated in the image processing step, both with and without preprocessing, we would like to have a set of sample orientation data which, when fitted, return the Péclet number corresponding to the distribution they were sampled from. To ensure this, we use rejection sampling from the marginal probability density function *Φ* at a selection of linearly spaced Péclet numbers 1 ≤ Pe ≤ 200, stopping when we have a set of angles *ϕ* which, when fitted, give a Péclet number 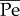 such that 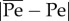. For each of these sets of angles, an M13 is then simulated with a given length *L*, and is placed at a point (*x*_0_, *y*_0_), randomly chosen with −0.5 μm ≤ *x*_0_, *y*_0_ ≤ 0.5 μm. An image of the M13 is then generated via (3.3), with *σ*_*x*_ given by following [25]. The intensity parameter *I*_0_ is chosen so that the image has a maximum intensity of 255, which corresponds to the maximum value an 8-bit unsigned integer can take, and hence the maximum intensity in the experimental images. The additive noise *B* in (3.3) is simulated by sampling from a normal distribution with a mean of 76.5 (30% of *I*_0_), and s.d. 5. These images are then put through both the image and Péclet fitting procedures, after which we are able to compare both the fitted angles 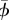 and fitted Péclet numbers 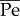. In order to evaluate the effectiveness of the preprocessing step, we analyse the same set of images twice, with and without the preprocessing step, and compare the results.

**Figure 5.**
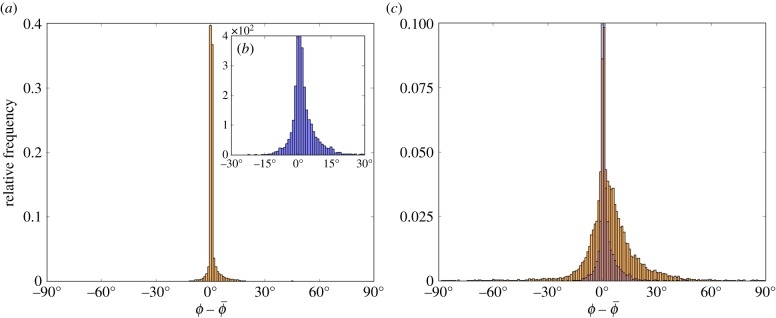
Relative frequency histograms of the error between the simulated M13 angles *ϕ* and fit angles 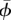, with 1° bin widths, for the analysis of the image fitting procedure. (*a*) The error distribution with the preprocessing step included, with the inset (*b*) being the same figure zoomed in for clarity. (*c*) The error distribution without the preprocessing step in orange, with the error with preprocessing overlaid in blue.

**Figure 6.**
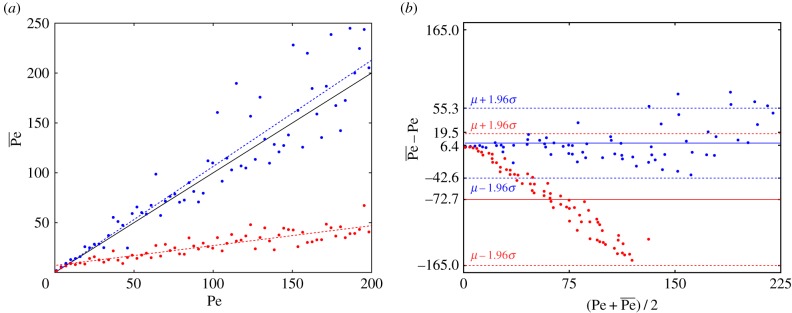
(*a*) The fitted Péclet number 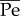 against the simulated Péclet number Pe, for analysis of the image fitting procedure. The blue dots show the fit with the preprocessing step, while the red dots show the fit without. The black line shows what would be perfect correspondence between Pe and 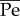, with the dotted lines being the line of best fit to the data. (*b*) The Bland–Altman plot testing the fitted data 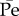 against Pe with preprocessing (blue) and without (red). The solid lines show the mean of the difference between 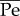 and Pe for each case, with the dotted lines being the 95% CI for the difference.

The number of images successfully analysed and the number of fit orientation angles 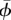 within 1° and 5° of simulated angles *ϕ* is shown in table 1, and the corresponding relative frequency histograms of the error between the simulated angles *ϕ* and fit angles 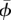 are shown in figure 5. It is clear when looking at these data that the inclusion of the preprocessing step improves the accuracy of the fit significantly. The Péclet numbers 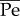 obtained through analysis of the fit orientation angles 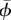 are then shown in figure 6. We see here that not including the preprocessing step results in a significant underestimation of the Péclet number for the flow, while the inclusion of the preprocessing step leads to results which accurately represent the simulated flows. We see from the least squares line of best fit that the image processing method provides good results, with a small increase in error for stronger flows (higher Péclet number). This is in agreement with the Bland–Altman plot, figure 6*b*. Here, we see a mean difference between Pe and 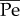 of 6.4 with the preprocessing step, and 72.7 without. Similarly, the standard deviation for the difference is 25 with preprocessing, compared with 47 without.

**Table 1.** Table showing the number and percentage of successfully fitted frames through the model-based image analysis procedure, and the number and percentage of fitted angles 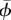 within 1° and 5° of the simulated angles *ϕ*. Here, step 1 and step 2 correspond to the analyses in §§4.1 and 4.2. A total of 12 060 images were analysed.

dataset	method	successfully fitted	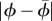	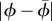
step 1	no preprocessing	11 674 (96.80%)	2337 (20.02%)	5684 (48.69%)
step 1	preprocessing	11 766 (97.56%)	9260 (78.70%)	10 840 (92.13%)
step 2	preprocessing	11 736 (97.31%)	9227 (78.62%)	10 779 (91.85%)

### 4.2. Step 2: error associated with the full analysis of Péclet number from a sample of orientation data

Having shown that the error in the image processing step is well contained with greater than 90% of fitted angles deviating from the simulated angles by less than 5°, we move on to look at the error associated with the full analysis of Péclet number from a sample of orientation data. We do this in the same way as in §4.1; however, instead of using rejection sampling to obtain a sample with the required Péclet number, we take a single sample of 180 angles *ϕ* from the marginal probability distribution *Φ* at each Pe. This should give insight into the accuracy of the full analysis on experimental images, with additional error being introduced through the generation of the orientation sample.

**Figure 7.**
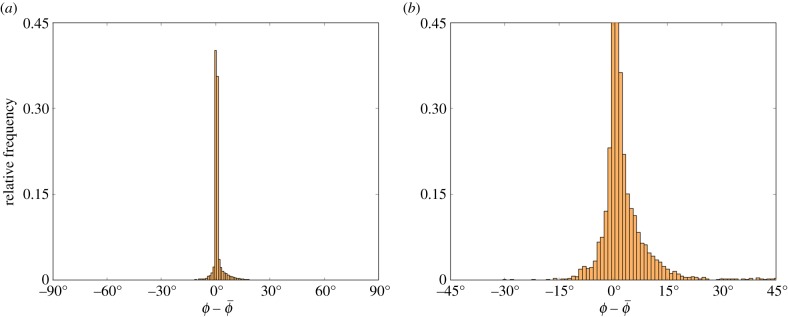
Relative frequency histograms of the error between the simulated M13 angles *ϕ* and fitted angles 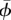, with 1° bin widths, for the full analysis. (*a*) The full error distribution with figure (*b*) zoomed in for clarity.

The images successfully analysed, along with the number of fitted orientation angles 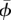 within 1° and 5° of the simulated angles *ϕ*, are again shown in table 1, with the corresponding relative frequency histograms of the error between *ϕ* and 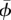 shown in figure 7. We see very similar results to that of step 1, which is to be expected as we have not changed the image analysis portion of the methods, which is independent of angle distribution.

**Figure 8.**
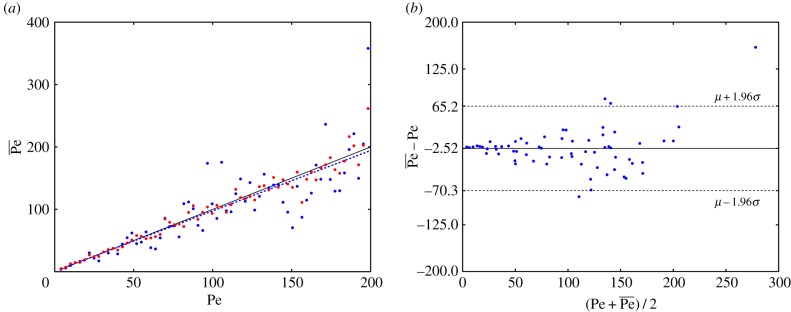
(*a*) The fitted Péclet number 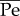 against the simulated Péclet number Pe for the full analysis. The blue dots show the fit after the image analysis has been carried out, while the red dots show the calculated Péclet number assuming the image analysis step is perfect. The black line shows what would be perfect correspondence between Pe and 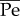, while the dotted blue line is the line of best fit to the data. (*b*) The Bland–Altman plot testing the fitted data 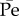 against Pe. The solid lines show the mean of the difference between 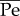 and Pe, with the dotted lines being the 95% confidence interval for the difference.

In figure 8, we plot the Péclet numbers obtained from fitting the angles 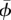. Included in this figure are the Péclet numbers given by fitting to the sampled angles (assuming a perfect image analysis method), where we can see the deviation about what would be perfect correspondence to the flow, which is a result of the restricted sample size in the simulations, and analogous to the error from having a restricted sample size in the related experiments. Here, the Bland–Altman plot, figure 8*b*, shows a mean difference between Pe and 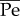 of 2.52, with the standard deviation of the difference being 35. We see here that, despite this additional error, and the error from the image processing procedure in step 1, we can reliably calculate the Péclet number relating to a given flow.

## 5. Calculation of Péclet number from experimental image data

**Figure 9.**
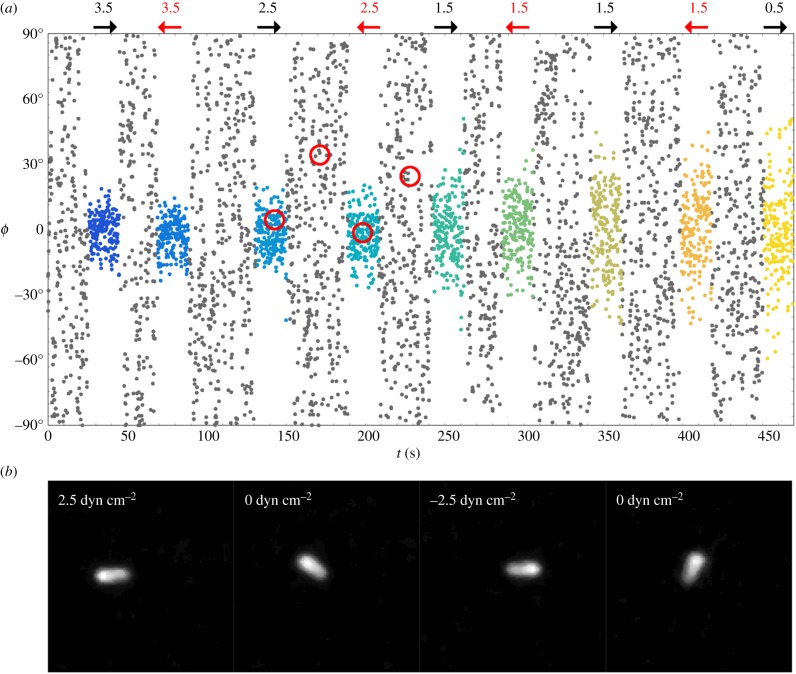
(*a*) Plot showing the angles *ϕ* obtained by fitting to the raw image data of Lobo *et al.* [11]. Here, the new analysis data have been presented in the style of Lobo *et al.* [11] for ease of comparison. Each grey dot represents an image where there is no nominal WSS, while the nominal WSS for each other colour is written above the figure, with an arrow indicating the direction of flow. (*b*) A selection of experimental images for the circled frames.

**Figure 10.**
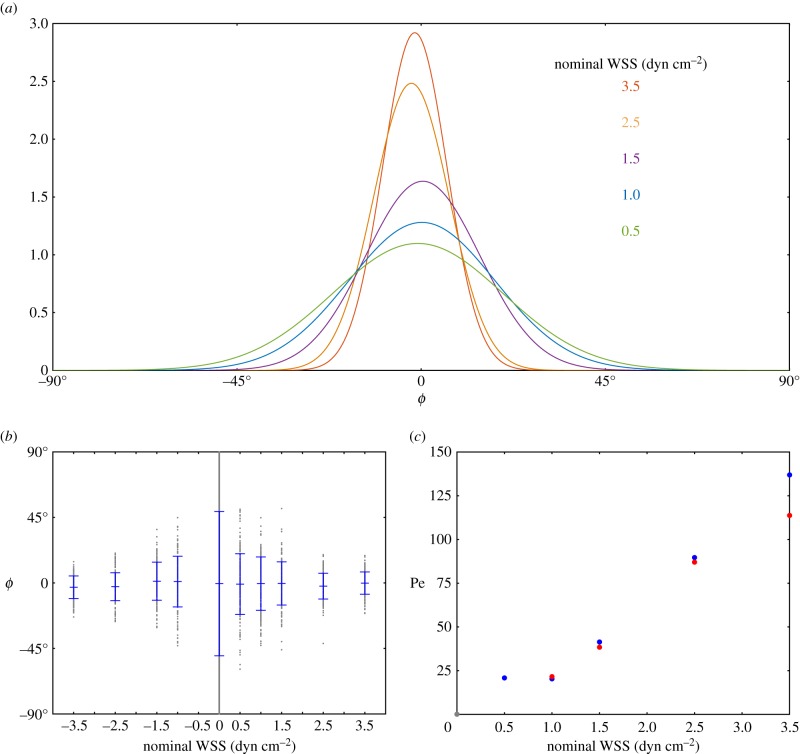
(*a*) Distribution of the angles *ϕ*, with lines showing a normalized Gaussian fit to the data. (*b*) Angle *ϕ* against nominal WSS. Here, the blue marks show the mean angle for each value of WSS, and the blue lines show 1 s.d. above and below the mean. (*c*) Here, the approximated Péclet number is shown against nominal WSS. The blue and red dots represent flow in the positive and negative directions, respectively, while the grey dot represents the case of zero applied flow.

The angles *ϕ* obtained from fitting the full series of raw image data from Lobo *et al.* [11] are shown in figure 9. Each point represents a single frame, with the corresponding experimentally applied nominal WSS, and direction, shown above the plot. Additionally, red circles show the location of four characteristic images, which are displayed at the bottom of the figure. It is clear by eye, before doing any in-depth analysis, that, when flow is applied, there is a strong biasing of the distribution of the M13 angle *ϕ* towards the direction of flow, and that this biasing effect is more pronounced the greater the nominal WSS. This is in agreement with the more detailed analysis shown in figure 10*a*,*b*. In figure 10*a*, we have fitted a normalized Gaussian model to the data for individual flow rates, combining data from flows of the same magnitude in different directions. It is clear from these figures that, as the nominal WSS increases, the probability that the M13 is aligned with the flow (towards *ϕ* = 0°) increases, with the standard deviation of the angles about *ϕ* = 0° decreasing. As expected, the flow direction does not have an impact on the distribution of the M13, as can be seen in figure 10*b*. Finally, we plot the estimated Péclet number for the flow in figure 10*c*, where it is clear that, with increased nominal WSS, we have fitted a larger Péclet number. We note that the Péclet number calculated for the 0.5 dyn cm^−2^ flow appears to be larger than expected. We believe that this is due to the fact that the flow lies outside the sensitivity range of the M13 in the experiments; a longer M13 would have more sensitivity to lower levels of WSS. This assertion is discussed in more detail in §6. We also see here that there is a slight discrepancy between the fit for the flows in the positive direction (blue) and that for the negative direction (red). This is to be expected from the statistical nature of the fit owing to the Fokker–Planck model, and we also expect some difference due to the fact that the collagen IV surface is not completely flat, leading to slight changes in flow behaviour in different directions. We believe that the fits in each direction are close enough to give credence to the viability of the fitting procedure.

## 6. Conclusion

It has recently been shown that a biological microrod (M13) can act as a WSS sensor [11] through flow-induced changes to its tethered Brownian motion. We have now developed and presented the first mechanistically rational analysis of this novel assay. This modelling and measurement framework consists of two steps, as follows.

(1) *Dynamics of a tethered Brownian fibre*. Here, we have modelled the rotational Brownian dynamics of a tethered Brownian fibre system under homogeneous unidirectional shear flow. Given experimentally calculated orientation data for an M13 under flow, the modelled orientation probability distribution for the M13 allows the calculation of a Péclet number for the flow, and hence a measure of the WSS over a biologically relevant surface.
(2) *Model-based image analysis*. To complement the mathematical modelling of the Brownian dynamics, we have developed a rigorous and extensible framework for the analysis of a set of experimental images. We have tackled the inverse problem of image formation, the solution to which allows the accurate and reliable calculation of the M13 location in a heavily diffracted image. This framework allows the swift, accurate and automated calculation of orientation data from experimental image data.

### 6.1. Comparison with previous work

We have applied this model to the problem of calculating WSS, validating against the work of Lobo *et al.* [11]. The present study differs from the previous analysis in that we have developed a principled and extensible framework based on modelling and statistics as opposed to small-angle approximations with pixel intensity thresholding and geometric operations to estimate M13 length and position calibrated with the experimental results. In analysing the same data, we have introduced the concepts of model-based image analysis and have tackled the inverse problem of image formation in order to locate the M13 in a series of experimental images. We believe that this approach to image analysis allows us to have more faith in the results over more traditional image analysis techniques. This is not only because of the inclusion of the physics of image formation in the underpinning model, but also because of the statistical framework for modelling the Brownian motion of the M13 which enables multiple sources of error to be considered in the analysis. The techniques introduced here also offer the advantage of being completely automated once set up; there is no manual component unlike many other methods, which allows the analysis of much larger quantities of data than would have been previously possible.

### 6.2. Applications and future extensions

We have shown that the combination of the fluid dynamic modelling of a tethered M13, together with the model-based image analysis of the experimental images, can produce an estimated Péclet number for the flow, which quantifies the relative importance of shear-driven and Brownian effects in the flow. Through simulations, we have produced an estimation of the accuracy of the model, and have shown that this method can reliably produce biologically relevant results. The methods can also be tailored to detect particular types of flow. The material time constant of Bird *et al.* [29] is calculated for the flow in this paper as 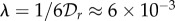 s, which is below the 0.1 s frame interval of the experiments of Lobo *et al.* [11]. In the analysis of other flows, the sampling rate must remain greater than the associated time constant to ensure the independent sampling of M13 positions in time. It is also of interest to note that rotational diffusion scales with length as 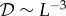, so small changes in M13 length have a large impact on the rotational diffusion coefficient, and hence Péclet number. The impact of this is that M13 engineered to be slightly longer will have a smaller diffusion coefficient and hence enables the detection window to be extended to lower shear rates; slightly shorter M13 will have a larger diffusion coefficient, hence enabling the detection window to be extended to higher shear rates—with the caveat that, for orientation to be detected, diffraction associated with the emission wavelength places a lower limit on M13 length.

The theory in this paper provides methods for calculating the Péclet number (as a proxy for WSS) on a flat surface through imaging of a tethered M13. The extensibility of the presented framework means that it will be possible to modify the fluid dynamic modelling (§2), together with added information from multi-plane imaging, to estimate the shear stress over more biologically relevant surfaces *in vivo*, e.g. over the endothelial cell lining of a blood vessel. The rotational Péclet number is proportional to the product of the shear rate 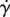 and local viscosity *μ*, i.e. the WSS (knowledge of the viscosity would then provide the value of the shear rate). Under the assumption of Newtonian rheology, uncertainty regarding the viscosity of a biological fluid is not problematic for the estimation of WSS because the Péclet number is determined precisely by WSS, temperature and the geometric properties of the phage. Given that the last two quantities are known accurately, this allows for highly reliable calculation of WSS in Newtonian fluids.

One particular application of this method would be in calculating WSS in the vasculature. While blood is known to behave as a non-Newtonian fluid macroscopically, on the microscopic length scale of M13 the relevant surrounding fluid is that of blood plasma, which behaves as a Newtonian fluid in shear flow [30]. It may, however, be of interest in future to characterize how the advective and diffusive terms in the model are modified by the inclusion of non-Newtonian rheology. Non-Newtonian properties may include, for example, shear-thinning or viscoelasticity. In the case of shear-thinning, we suggest that the shear rate in the vessel is likely to change on a length scale of an order of magnitude longer than the phage. Therefore, the advection and diffusion of the phage can be well approximated by a Newtonian model. As discussed above, the Péclet number provides information about the WSS, so it is not necessary to know about the viscosity directly. For the case of fluids which have a significant viscoelastic rheology on the microscopic scale of M13, for example mucus, the fluid dynamic framework would need to be extended to incorporate such effects.

While our model of M13 as a rigid rod-like structure is a rational first approximation, it could be improved through coupling the fluid mechanics calculations with an elastohydrodynamic model for M13 bending, for example by using the model of Montenegro-Johnson *et al.* [31].

Additionally, regarding the model-based image analysis, if we were able to accurately measure the optical diffusion in a given experimental set-up, and relate this to the PSF model (3.2), we should be able to obtain the full three-dimensional reconstruction of the M13 location, which would then allow the use of the full probability density function *ψ*, rather than the marginal probability density function, *Φ*, as obtained in §2. We would expect good results in the full three-dimensional case, even if the surface is not perpendicular to the imaging plane provided there was some knowledge about the surface topography. Topographic information could be quantified, for example via the multiple imaging plane set-up of Dalgarno *et al.* [32]. Such experimental techniques would provide knowledge of the instantaneous geometry and potential wall movements due to elasticity, pulsatile flow or other fluctuations. These effects could then be coupled with the fluid mechanics calculations via the boundary conditions to increase the applicability of the model *in vivo*.

Throughout this work, we have considered the analysis of a single M13, and the calculation of the Péclet number for the system. While this is a good method for understanding the dynamics of a locally spatially homogeneous topography, problems arise when there is a significant change of topology in space. In addition to improving the optical approaches to the problem, the analysis in this method could be extended to consider data from a set of spatially distributed M13 in order to better understand the behaviour of flow across complex geometries.

While in this work we have considered only the calculation of surface shear stress, we believe the ‘ethos’, as well as the techniques, developed here could have wider applications in the fields of microscale biology and image analysis. Of great interest is the application of the model-based image analysis techniques to experimental data of motile cells such as sperm. We believe that these techniques will be able to provide great insight into a variety of topics, from subcellular structures to flagellated swimming cells such as sperm, and will have the potential for wide-ranging impact in fields such as fertility and animal husbandry.

## Endnote

1 Here, we have shown all angles in degrees for consistency with the results presented in Lobo *et al.* [11]. However, all calculations have been performed in radians.

## Data accessibility

All the code for this project, and data for the generation of figures, can be accessed at https://github.com/meuriggallagher/phage.

## Appendix A. Derivation of the advection–diffusion equation for a tethered fibre

The flux of the probability density function *ψ*(*θ*, *ϕ*, *t*) is given by , where  is the rate of change of ***d*** due to the combination of hydrodynamic and Brownian rotations. Denoting by  the torque-free angular velocity of the particle induced by the shear flow, then the rate of change of ***d*** under rigid body rotation is

A 1

In the presence of the shear flow, the hydrodynamic torque ***T***^H^ produced by a fibre rotating with angular velocity ***ω*** is given by

A 2

where  is the rotational resistance matrix about the origin, taking into account the effect of the plane boundary. The *θ*-dependence is a consequence of the boundary effect.

Following [22], the Brownian torque on a suspension is given by

A 3

where *k* is the Boltzmann constant and *T* is the absolute temperature. Torque balance ***T***^H^ + ***T***^B^ = 0 then yields

A 4

By rearranging we have

A 5

where  is the rotational diffusion matrix and  is the rotational advection vector.

## Appendix B. Numerical solution of the advection–diffusion equation

The diffusion tensor  for an axisymmetric body can be written,

B 1

The advective term ***α*** is given by

B 2

where ***α*** = ***ω*** × ***d***.

In component form, equation (2.8) can be written as

B 3

where we have assumed that *ψ* is time independent, and dropped dashes on dimensionless variables for brevity. The system is solved numerically via a finite difference method to give an approximate solution [*ψ*_*ij*_] ≈ *ψ*(*θ*_*i*_, *ϕ*_*j*_) (*i* = 1, …, 100, and *j* = 1, …, 100) on the domain 0° < *θ* < 90°, 0° ≤*ϕ* < 360° for a given Péclet number Pe.

## References

[1] Reneman RS, Arts T, Hoeks APG 2006 Wall shear stress—an important determinant of endothelial cell function and structure—in the arterial system *in vivo*. J. Vasc. Res. 43, 251–269. (10.1159/000091648) 16491020

[2] Fisher AB, Chien S, Barakat AI, Nerem RM 2001 Endothelial cellular response to altered shear stress. Am. J. Physiol. Lung C. 281, L529–L533. 10.1152/ajplung.2001.281.3.L529 11504676

[3] Nge PN, Rogers CI, Woolley AT 2013 Advances in microfluidic materials, functions, integration, and applications. Chem. Rev. 113, 2550–2583. (10.1021/cr300337x) 23410114 PMC3624029

[4] El-Ali J, Sorger PK, Jensen KF 2006 Cells on chips. Nature 442, 403–411. (10.1038/nature05063) 16871208

[5] Lee SJ, Kim S 2009 Advanced particle-based velocimetry techniques for microscale flows. Microfluid. Nanofluid. 6, 577–588. (10.1007/s10404-009-0409-6)

[6] Große S, Schröder W 2007 Mean wall-shear stress measurements using the micro-pillar shear-stress sensor MPS^3^. Meas. Sci. Technol. 19, 015403 (10.1088/0957-0233/19/1/015403)

[7] Brücker C, Spatz J, Schröder W 2005 Feasability study of wall shear stress imaging using microstructured surfaces with flexible micropillars. Exp. Fluids 39, 464–474. (10.1007/s00348-005-1003-7)

[8] Poelma C, Vennemann P, Lindken R, Westerweel J 2008 In vivo blood flow and wall shear stress measurements in the vitelline network. Exp. Fluids 45, 703–713. (10.1007/s00348-008-0476-6)

[9] Sugii Y, Okuda R, Okamoto K, Madarame H 2005 Velocity measurement of both red blood cells and plasma of *in vitro* blood flow using high-speed micro PIV technique. Meas. Sci. Technol. 16, 1126 (10.1088/0957-0233/16/5/011)

[10] Smith ML, Long DS, Damiano ER, Ley K 2003 Near-wall *μ*-PIV reveals a hydrodynamically relevant endothelial surface layer in venules in vivo. Biophys. J. 85, 637–645. (10.1016/S0006-3495(03)74507-X) 12829517 PMC1303118

[11] Lobo DP et al. 2015 Direct detection and measurement of wall shear stress using a filamentous bio-nanoparticle. Nano Res. 8, 3307–3315. (10.1007/s12274-015-0831-x) 27570611 PMC4996322

[12] Khalil AS, Ferrer JM, Brau RR, Kottmann ST, Noren CJ, Lang MJ, Belcher AM 2007 Single m13 bacteriophage tethering and stretching. Proc. Natl Acad. Sci. USA 104, 4892–4897. (10.1073/pnas.0605727104) 17360403 PMC1829235

[13] Chen P-Y, Ladewski R, Miller R, Dang X, Qi J, Liau F, Belcher AM, Hammond PT 2013 Layer-by-layer assembled porous photoanodes for efficient electron collection in dye-sensitized solar cells. J. Mater. Chem. A 1, 2217–2224. (10.1039/C2TA00771A)

[14] Ghosh D, Lee Y, Thomas S, Kohli AG, Yun DS, Belcher AM, Kelly KA 2012 M13-templated magnetic nanoparticles for targeted in vivo imaging of prostate cancer. Nat. Nanotechnol. 7, 677–682. (10.1038/nnano.2012.146) 22983492 PMC4059198

[15] Carr-Smith J et al. 2015 Polymerase chain reaction on a viral nanoparticle. ACS Synth. Biol. 4, 1316–1325. (10.1021/acssynbio.5b00034) 26046486

[16] Kim J et al. 2017 Monitoring the orientation of rare-earth-doped nanorods for flow shear tomography. Nat. Nanotechnol. 12, 914–919. (10.1038/nnano.2017.111) 28650436

[17] Pacheco-Gómez R et al. 2011 Detection of pathogenic bacteria using a homogeneous immunoassay based on shear alignment of virus particles and linear dichroism. Anal. Chem. 84, 91–97. (10.1021/ac201544h) 22017566

[18] Strand SR, Kim S, Karrila SJ 1987 Computation of rheological properties of suspensions of rigid rods: stress growth after inception of steady shear flow. J. Non-Newt. Fluid Mech. 24, 311–329. (10.1016/0377-0257(87)80044-7)

[19] Ota S, Li T, Li Y, Ye Z, Labno A, Yin X, Alam M-R, Zhang X 2014 Brownian motion of tethered nanowires. Phys. Rev. E 89, 053010 (10.1103/PhysRevE.89.053010) 25353883

[20] Schindelin J, Rueden CT, Hiner MC, Eliceiri KW 2015 The ImageJ ecosystem: an open platform for biomedical image analysis. Mol. Reprod. Dev. 82, 518–529. (10.1002/mrd.22489) 26153368 PMC5428984

[21] Matlab. 2017 *Version 9.2 (R2017a)*. Natick, MA: The MathWorks Inc.

[22] Kim S, Karrila SJ 1991 Microhydrodynamics: principles and selected applications. London, UK: Butterworth-Heinemann.

[23] Smith DJ 2017 A nearest-neighbour discretisation of the regularized stokeslet boundary integral equation. (https://arxiv.org/abs/1704.09022)

[24] Ainley J, Durkin S, Embid R, Boindala P, Cortez R 2008 The method of images for regularized stokeslets. J. Comp. Phys. 227, 4600–4616. (10.1016/j.jcp.2008.01.032)

[25] Zhang B, Zerubia J, Olivo-Marin J-C 2007 Gaussian approximations of fluorescence microscope point-spread function models. Appl. Opt. 46, 1819–1829. (10.1364/AO.46.001819) 17356626

[26] The Numerical Algorithms Group (NAG). The NAG toolbox for Matlab. Oxford, UK: NAG.

[27] Arce GR 2005 Nonlinear signal processing: a statistical approach. New Jersey, NJ: Wiley.

[28] Daniel WW 1990 Applied nonparametric statistics. The Duxbury Advanced Series in Statistics and Decision Sciences Boston, MA: PWS-Kent Publ.

[29] Bird RB, Armstrong RC, Hassager O 1977 Dynamics of polymeric liquids, vol. 2 New York, NY: John Wiley & Sons.

[30] Brust M, Schaefer C, Doerr R, Pan L, Garcia M, Arratia PE, Wagner C 2013 Rheology of human blood plasma: viscoelastic versus newtonian behavior. Phys. Rev. Lett. 110, 078305 (10.1103/PhysRevLett.110.078305) 25166417

[31] Montenegro-Johnson TD, Gadêlha H, Smith DJ 2015 Spermatozoa scattering by a microchannel feature: an elastohydrodynamic model. R. Soc. open sci. 2, 140475 (10.1098/rsos.140475) 26064617 PMC4448824

[32] Dalgarno PA, Dalgarno HIC, Putoud A, Lambert R, Paterson L, Logan DC, Towers DP, Warburton RJ, Greenaway AH 2010 Multiplane imaging and three dimensional nanoscale particle tracking in biological microscopy. Opt. Express 18, 877–884. (10.1364/OE.18.000877) 20173908

